# Implementation of the multielectrode radiofrequency-balloon in real-world clinical practice—operator learning curve and procedural outcome at a high-volume center

**DOI:** 10.3389/fcvm.2023.1208250

**Published:** 2023-11-14

**Authors:** Maura M. Zylla, Lydia Starrach, Ann-Kathrin Rahm, Dierk Thomas, Norbert Frey, Patrick Lugenbiel

**Affiliations:** ^1^ Department of Cardiology, Medical University Hospital, Heidelberg, Germany; ^2^ HCR (Heidelberg Center for Heart Rhythm Disorders), Medical University Hospital, Heidelberg, Germany; ^3^ DZHK (German Center for Cardiovascular Research), Partner Site Heidelberg/Mannheim, Heidelberg, Germany

**Keywords:** atrial fibrillation, catheter ablation, pulmonary vein isolation, radiofrequency balloon, operator learning curve

## Abstract

**Background:**

The novel multielectrode radiofrequency (RF) balloon catheter (HELIOSTAR™, Biosense Webster) is a new technology for pulmonary vein isolation (PVI) in atrial fibrillation (AF), combining RF-ablation and 3D-mapping visualization with the concept of a “single-shot”-ablation device. This study evaluates the operator learning curve und procedural outcome during implementation of the multielectrode RF-balloon at a high-volume center.

**Methods:**

The first 40 patients undergoing PVI by multielectrode RF-balloon catheter at Heidelberg University Hospital were included in this prospective study. Procedural outcome was analyzed over the course of increasing experience with the device.

**Results:**

157/157 pulmonary veins (PVs) were successfully isolated with the RF-balloon catheter, in 73.2% by a single RF-application. Median time to isolation (TTI) was 11.0 s (Q1 = 8.0 s; Q3 = 13.8 s). Median procedure time was 62.5 min (Q1 = 50.0 min; Q3 = 70.5 min). LA-dwell time was 28.5 min (Q1 = 23.3 min; Q3 = 36.5 min). Median fluoroscopy duration was 11.6 min (Q1 = 10.1 min; Q3 = 13.7 min). No serious procedure-related complications were observed, apart from one case of unclear, post-procedural acute-on-chronic kidney injury. With increasing operator experience, an additional reduction in procedure duration was observed.

**Conclusion:**

Rapid implementation of a “single shot”-ablation device combining RF-ablation and 3D-mapping can be achieved with high acute procedural efficacy and safety at a high-volume center. Previous experience with “single-shot” ablation devices may be advantageous for time-efficient introduction of the novel RF-balloon catheter into clinical practice.

**Clinical Trial Registration:**

ClinicalTrials.gov; Identifier NCT0560361.

## Introduction

Catheter ablation has emerged as a widely established interventional therapy for rhythm control in atrial fibrillation (AF). Pulmonary vein isolation (PVI) is the cornerstone of AF ablation, targeting mechanistic triggers located at the PV-ostia (1). Circumferential “point-by-point” PVI with radiofrequency (RF) energy delivery via linear ablation catheters and cryoballoon (CB) ablation using over-the-wire “single-shot” ablation catheters are commonly used methods. Whereas both techniques are associated with comparable success rates and safety, they offer different advantages and are associated with different potential—albeit rare—complications (2).

The novel multielectrode RF-balloon catheter (HELIOSTAR™) (Biosense Webster, Johnson & Johnson, Irvine, CA, USA) strives to combine the advantages of these two methods. It enables delivering tailored local RF-energy applications and visualization in the CARTO® 3D-mapping system (Biosense Webster) while employing the concept of “single-shot”-ablation device, with the aim of reducing procedure times and complexity of operator training. Additionally, the multielectrode RF-balloon catheter offers the potential to reduce radiation dose and contrast application by verifying adequate contact with the PV-ostia via impedance and temperature measurements. Previous multicenter studies showed safety and high acute efficacy of PVI with the novel RF-balloon catheter (3, 4).

Due to the increasing prevalence of AF and, thus, growing demand for interventional AF-therapies, a time-efficient introduction of new technologies into clinical routine is of utmost importance. This study evaluates procedural parameters during implementation of the RF-balloon catheter at a high-volume AF-ablation center, as well as the operator learning curve in an operator experienced in CB-ablation.

## Methods

The aim of this prospective, observational analysis was to evaluate procedural parameters, as well as the operator learning curve, during implementation of the multielectrode RF-balloon catheter in real-world clinical practice at a high-volume center. This study was performed in accordance with the principles of the Declaration of Helsinki. It has been approved by local ethics committee (Study Identifier: S-815/2021) and registered on ClinicalTrials.gov (Identifier Number: NCT05603611).

### Study population

Data from 40 consecutive patients from a high-volume ablation center (^∼^800 ablation procedures/year, >350 PVIs/years) undergoing ablation by the novel RF-balloon catheter were analyzed. Procedures were performed by an operator proficient in CB-ablation (experience of >600 procedures) but without prior experience with the investigated device. Patients were recruited from regular clinical routine and presented to the Heidelberger University Hospital with an indication for AF ablation. Inclusion criteria were age ≥18 years, ability to provide informed consent and at least one episode of ECG-documented paroxysmal or persistent AF. Exclusion criteria were history of prior AF ablation, left atrial thrombus, irregular PV-anatomy in pre-procedural transesophageal echocardiography (TOE) which was suspected to be inaccessible for standard PVI protocols or contraindication for peri-procedural anticoagulation therapy. Demographic and clinical baseline parameters were systematically recorded at recruitment. Procedures were performed between 10/2021 and 07/2022.

### Index procedure and peri-procedural management

The procedure was performed according to guidelines and the center’s standards in conjunction with the manufacturer’s instructions as to handling of the multielectrode RF-balloon catheter.

**Figure 1:**
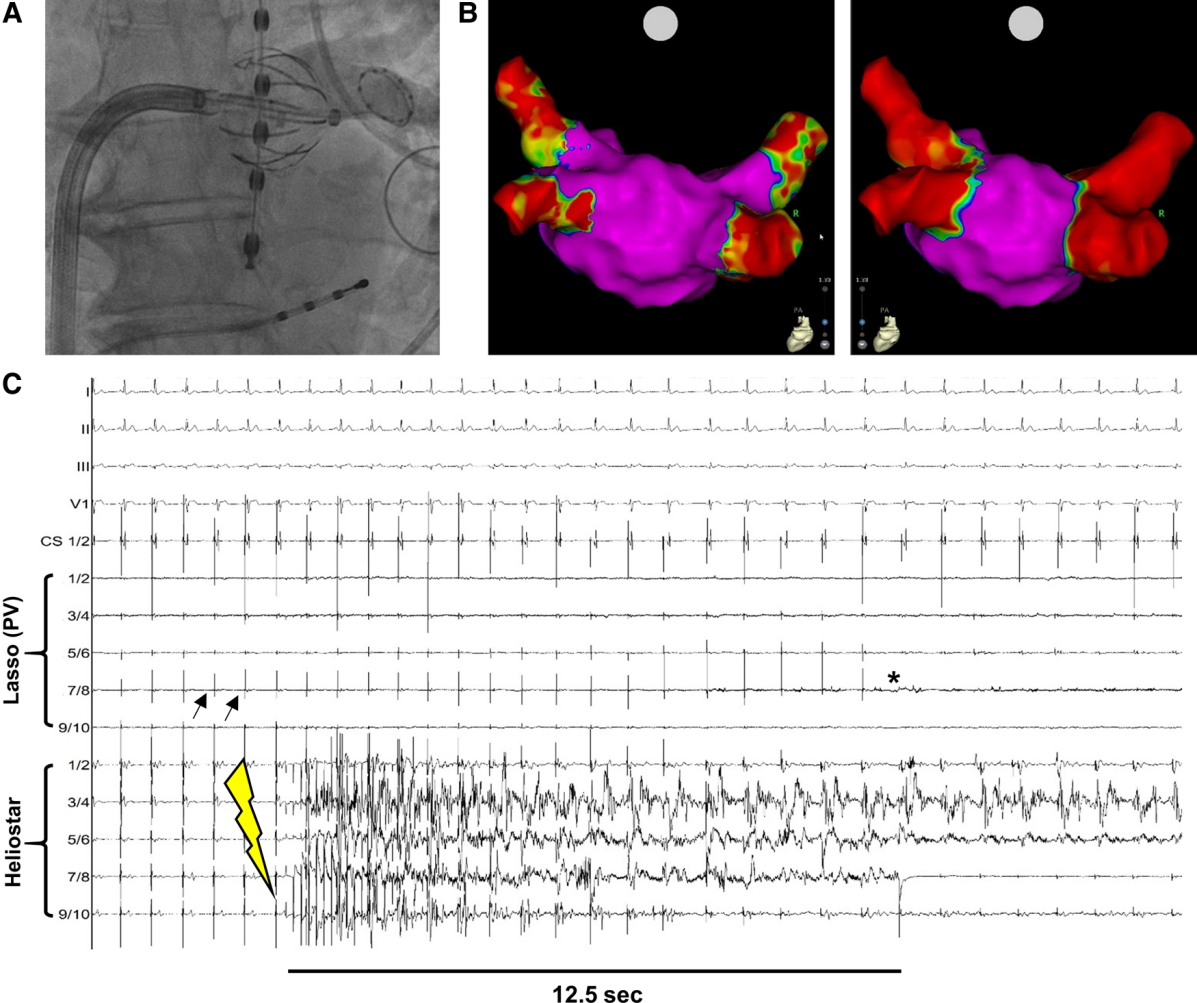
Example of PVI with the novel multielectrode RF-balloon catheter. (**A**) Fluoroscopy-guided angiography to verify contact between HELIOSTAR™ and left superior PV-ostium. The oesophageal temperature probe is positioned in direct proximity to the ablation catheter. (**B**) Left atrial electroanatomical, bipolar map before (*left*) and after (*right*) PVI by HELIOSTAR™. Note the clearly demarcated ostial lesions after ablation. (**C**) Example tracing during PVI with HELIOSTAR™. Black arrows indicate PV-signals in the LASSOSTAR™ catheter, with entrance block after PVI (*). Yellow flash-symbol indicates start of ablation. PVI is achieved after 12.5 s. PV, pulmonary vein.

All patients received oral anticoagulation therapy for at least 3 weeks prior to the procedure. Patients with CHA_2_DS_2_-VASc-Scores of ≥1 (men) or ≥2 (women) additionally underwent pre-procedural TOE to rule out intracardiac thrombus prior to PVI. Patients receiving NOAC were advised to pause anticoagulation medication at least 12 h (in case of NOAC b.i.d) prior to the ablation procedure. Vitamin K antagonists were continued with a target INR of 2.0–2.5 at the time of procedure. The right femoral vein was used as preferred access site. A quadripolar diagnostic catheter was placed in the coronary sinus. Temperature in the surrounding tissues was monitored by an esophageal probe. During the procedure, heparin was administered to achieve an activated clotting time (ACT) between 300 and 400 s. Pre-ablation 3D-mapping was performed with the help of the LASSO-NAV™ catheter (Biosense Webster) due to non-availability of the navigational LASSOSTAR™-catheter during the limited market release period. Prior to RF-energy delivery, local PV-activity was monitored after introducing the LASSOSTAR™ catheter (non-navigational) into the PV-ostium. After inflation of the RF-balloon catheter, PV-angiography was performed to additionally assess co-axiality and optimize occlusion before PV-ablation (Figure 1A). Target values for optimal local contact prior to energy application were an inflation index of >0.8, temperature below 31°C and an impedance of >100 Ω with minimum variation across all electrodes. These were in accordance with the optimized procedural workflow for the RF-balloon catheter (5). RF-energy of 15 Watts was delivered for 20 s at the posterior wall and 60 s at the other segments. Target impedance drop during lesion creation was >12 Ohm. During ablation of the right pulmonary veins phrenic nerve function was monitored by diaphragmatic motion during phrenic pacing. Prior to energy application at the septal PVs, critically close anatomical location of the phrenic nerve was excluded by pacing via the anteriorly located electrodes of RF-balloon catheter. Local energy application was stopped in case of sudden temperature rise >2°C in the esophageal probe or loss of phrenic capture.

Acute PV-reconnection was excluded by a 3D-remap or assessment of local electrograms in the LASSOSTAR™ catheter at the end of the procedure. The center-specific protocol did not include a prolonged intraprocedural waiting time after PVI nor adenosine application for assessment of dormant PV conduction. Echocardiography was performed routinely after the procedure and repeated after 24 h to exclude pericardial effusion. Anticoagulation was resumed immediately after the procedure. Endoscopy was only performed in patients with clinical suspicion of oseophageal lesions or atrio-oesophageal fistula.

### Endpoints and follow-up

Procedural endpoints, including overall procedure duration, radiation exposure, number and duration of energy applications and time to PV-isolation (TTI) were assessed. Additional efficacy endpoints investigated in an exploratory manner included rate of successful PVI, short-term arrhythmia recurrence during index hospital stay and arrhythmia recurrence >3 months after the index procedure. The first three months after the index procedure were regarded as the “blanking period” in analogy with previous AF-ablation studies. Arrhythmia recurrence was defined as recurrence of any symptomatic and documented episode of AF or left atrial arrhythmia lasting >30 s. Occurrence of typical atrial flutter during the follow-up period was recorded but not included in the efficacy analysis due to the underlying pathophysiological mechanism independent from the index procedure. Safety endpoints for exploratory analysis included major and minor procedure-associated complications. For clinical follow-up, patients were subsequently included in a prospective registry with outpatient follow-up visits after 3 months and telephone-based follow-ups at 6 and 12 months after the index procedure. Additionally, any unscheduled outpatient or in-hospital visits for cardiovascular reasons were recorded for endpoint evaluation.

Repeat ablations were performed using the CARTO® 3D-mapping system (Biosense Webster) in order to detect PV re-connection and characterize atrial substrate. In cases of atrial tachycardia, both an activation map of the arrhythmia and a substrate map were created. Tachycardia was induced in patients presenting in sinus rhythm who had ECG-documented regular atrial tachycardia or atrial flutter. PV re-isolation or specific ablation according to arrhythmia mechanism was performed by RF-ablation via a linear ablation catheter (Thermocool Smarttouch®, Biosense Webster).

### Statistical analysis

In order to evaluate the operator learning curve, procedural endpoints were analyzed over the course of increasing experience with the device and compared between the subgroup of the first ten patients and the last ten patients included. Endpoints were analyzed in an observational manner. Due to the exploratory character of this analysis, the *P*-values are of descriptive nature and no adjustment for multiple testing was applied. For descriptive analyses, continuous variables are reported as median with inter-quartile range (Q1, Q3). Comparisons between groups were performed using Fisher’s exact test in case of categorical variables or Mann–Whitney-*U* test in case of continuous variables. Correlations between outcome variables were calculated as Pearson correlation coefficients and corresponding confidence intervals. *P*-values <0.05 were denoted as statistically significant. The statistical analysis was performed using SPSS-version 29.0.

## Results

### Patient cohort undergoing PVI with the novel multielectrode RF-balloon catheter

The majority of patients in this cohort were male, displayed preserved ejection left ventricular fraction and mildly dilated left atria (Table 1). Most patients had been diagnosed with paroxysmal AF, whereas 32.5% of patients suffered from persistent AF. Arterial hypertension was the most common cardiac co-morbidity. In 15.0% of patients, pharmacological rhythm control strategies had been previously attempted but had not yielded sufficient symptom control (Table 1).

**Table 1:** Baseline parameters.

Baseline parameters	
Age, years, median (Q1;Q3)	64.5 (58.3;72.5)
Male, *n* (%)	29 (72.5)
Paroxysmal AF, *n* (%)	27 (67.5)
Persistent AF, *n* (%)	13 (32.5)
LA-diameter, mm, median (Q1;Q3)	44.5 (38.8;47.8)
LVEF >55%, *n* (%)	29 (72.5)
LVEF 45–54%, *n* (%)	6 (15.0)
LVEF 35–44%, *n* (%)	2 (5.0)
LVEF <35%, *n* (%)	3 (7.5)
Previous therapy with AAD, *n* (%)	6 (15.0)
CHA_2_DS_2_-VASc, median (Q1;Q3)	2.0 (1.0;3.0)
EHRA, median (Q1;Q3)	2.0 (2.0;2.8)
Hypertension, *n* (%)	33 (82.5)
Coronary artery disease, *n* (%)	8 (20.0)
BMI, kg/m^2^, median (Q1;Q3)	28.5 (26.3,30.9)

AAD, antiarrhythmic drugs; BMI, body mass index; EHRA, European Heart Rhythm Association; LA, left atrium; LVEF, left ventricular ejection fraction.

### Procedural and medium-term clinical outcome

In the first 40 patients treated with the multielectrode RF-balloon catheter at our center, 157/157 pulmonary veins (PVs) could be successfully isolated, including three cases with left common PV-ostia. In 115 PVs (73.2%) ablation was achieved by a “single-shot” RF-application. This includes single-shot PVI in PVs with measurable ectopic PV-activity, as well as PVs with no local activity at the time of ablation but at which procedural target values regarding RF-energy application were met. In PVs in which ectopic PV-activity could be detected via the LASSOSTAR™ or in the balloon electrodes prior to ablation (*n* = 66) (Figure 1C), median TTI was 11.0 s (Q1 = 8.0 s; Q3 = 13.8 s). Median procedure duration (“skin-to-skin”) was 62.5 min (Q1 = 50.0 min; Q3 = 70.5 min). This also included time to LA-access (median = 11.0 min; Q1 = 8.3 min; Q3:14.0 min) and mapping time (median = 9.0 min; Q1 = 7.0 min; Q3 = 10.0 min). Median LA-dwell time was 28.5 min (Q1 = 23.3 min; Q3 = 36.5 min) and median duration of RF-energy application was 5.0 min (Q1 = 4.0 min; Q3 = 6.8 min). A 3D-remap was performed in six cases (median duration = 8.0 min; Q1 = 6.0 min; Q3 = 10.5 min) (Figure 1B), in the other cases persistent post-procedural PVI was verified by real-time electrogram analysis with the help of the LASSOSTAR™ catheter. Median fluoroscopy duration was 11.6 min (Q1 = 10.1 min; Q3 = 13.7 min) and median fluoroscopy dose amounted to 3.1 Gyxcm^2^ (Q1 = 2.4 Gyxcm^2^; Q3 = 4.3 Gyxcm^2^).

RF-application had to be prematurely terminated at 13 PVs (8.3%) due to temperature rise in the oesophageal probe (maximum temperature rise to 47°C). At 4 PVs (2.5%), only segmental ablation was performed due to phrenic capture at safety pacing via the HELIOSTAR™-electrodes. In 9 PVs (5.7%), acute reconnection was observed resulting in re-ablation during the index procedure leading to additional RF-energy delivery. During 9 procedures (22.5%), intra-procedural troubleshooting was necessary due to technical issues with generator, sheath or mapping system causing procedural delays. Time for troubleshooting were included in the procedural and LA dwell times outlined above. There was no statistically significant difference in procedure duration between sexes (*P* = 0.192) or according to type of AF (*P* = 0.85), and no significant correlation with LA-diameter [correlation coefficient = 0.056, (CI = −0.260;0.362)]. Procedure duration showed a weak correlation with BMI [correlation coefficient = 0.432, (CI = 0.129;0.649)] and patients in the last quartile of the cohort had a slightly lower median BMI [27.7 kg/m^2^ (Q1 = 24.2 kg/m^2^; Q3 = 30.3 kg/m^2^)] than patients in the first quartile [29.0 kg/m^2^ (Q1 = 26.6 kg/m^2^; Q3 = 33.1 kg/m^2^)], *P* = 0.045).

Follow-up data from six months after the index procedure were available in 34 patients. Nine patients continued antiarrhythmic medication (26.5%), of which three patients received amiodarone (8.8%). Sixteen patients (47.1%) experienced arrhythmia recurrence and 5 patients (14.7%) underwent repeat ablation during the follow-up period. In two patients, persistent PV isolation was confirmed. In one of these patients, three distinct mechanisms of atrial flutter were diagnosed in the redo procedure and were treated by establishing an LA roof line, an anterior LA line as well cavotricuspid isthmus block. No arrhythmia was inducible after ablation. In the other patient a focal tachycardia originating from an inhomogeneous region near the ostium of the right superior PV was diagnosed in 3D mapping. The arrhythmia terminated and was no longer inducible after local ablation in this region. In the remaining three patients with arrhythmia recurrence, the reconnected veins were isolated, without the creation of additional ablation lines or ablation of complex fractionated atrial electrograms (CFAE). In one patient, the left superior PV was shown to be reconnected and re-isolated during the redo-procedure. In another patient re-connection at a left common ostium and both septal PVs was detected. In this patient, only segmental ablation had been performed at the septal PVs during the index procedure due to phrenic capture at safety pacing via the balloon electrodes prior to ablation. All veins could be successfully re-isolated during the redo-procedure. In the last patient undergoing a repeat ablation, reconnection of both septal PVs was shown with persistent isolation of the lateral PVs. In the index procedure, ablation of the right inferior PV had been terminated at <60 s, after establishing entrance block, due to temperature rise in the esophageal probe. The septal PVs could be re-isolated successfully in the redo-procedure.

In order to assess the role of disease progression for rhythm-associated outcome, we performed a subgroup analysis of patients according to type of AF (Table 2). Patients with persistent AF were characterized by larger LA diameters and lower LVEF (Table 2). However, median ejection fraction was preserved in both subgroups. Demographic baseline parameters, other co-morbidities and procedural endpoints did not differ between the two subgroups. The rate of arrhythmia recurrence was numerically higher in patients with persistent AF, however without reaching statistical significance. The majority of patients with paroxysmal AF and documented arrhythmia recurrence again developed AF as recurrent arrhythmia, whereas nearly a third of patients with persistent AF developed atrial tachycardia (Table 2).

**Table 2:** Subgroup analysis according to type of atrial fibrillation.

	Paroxysmal AF (*n* = 27)	Persistent AF (*n* = 13)	*P*-value
Baseline parameters			
Age, years, median (Q1; Q3)	64.0 (58.0;71.0)	66.0 (58.0;76.5)	0.441
Male, *n* (%)	20 (74.0)	9 (69.0)	1.000
LA-diameter, mm, median (Q1; Q3)	43.0 (37.0;46.0)	46.0 (45.0;51.0)	0.005
LVEF, %, median (Q1; Q3)	58.0 (55.0;60.0)	55.0 (44.0;56.5)	0.025
CHA_2_DS_2_-VASc, median (Q1; Q3)	2.0 (1.0;3.0)	3.0 (2.0;3.5)	0.055
Hypertension, *n* (%)	22 (81.5)	11 (84.6)	1.000
Coronary artery disease, *n* (%)	6 (22.2)	2 (15.4)	1.000
BMI, kg/m^2^, median (Q1; Q3)	27.6 (26.3,30.5)	29.4 (26.2,31.6)	0.345
Procedural parameters			
Procedure duration (Q1; Q3)	59,0 (49.0;71.0)	68,0 (58.5;71.0)	0.089
LA dwell time (Q1; Q3)	27.0 (23.0;35.0)	29.0 (25.0;39.5)	0.530
Fluoroscopy duration (Q1; Q3)	11.1 (9.8;13.3)	13.0 (11.3;16.5)	0.100
Duration energy application (Q1; Q3)	5.0 (4.0;6.0)	5.0 (4.0;8.5)	0.493
Clinical outcome (*n* = 34)			
Arrhythmia recurrence, *n* (%)	9 (33.3)	7 (53.8)	0.475
Atrial fibrillation, *n* (%)	8 (88.9)	5 (71.4)	
Atrial tachycardia, *n* (%)	1 (11.1)	2 (28.6)	
Repeat ablation, *n* (%)	4 (14.8)	1 (7.7)	0.673

AAD, antiarrhythmic drugs; BMI, body mass index; EHRA, European Heart Rhythm Association; LA, left atrium; LVEF, left ventricular ejection fraction.

In order to detect potential predictors for arrhythmia recurrence, baseline parameters between patients with and without arrhythmia recurrence at six months were compared. There was no statistically significant difference regarding age, sex, LA diameter, type of AF, LVEF, cardiovascular risk factors, BMI or rate of in-hospital recurrence between the two subgroups (Supplementary Table S1).

### Operator learning curve

**Figure 2:**
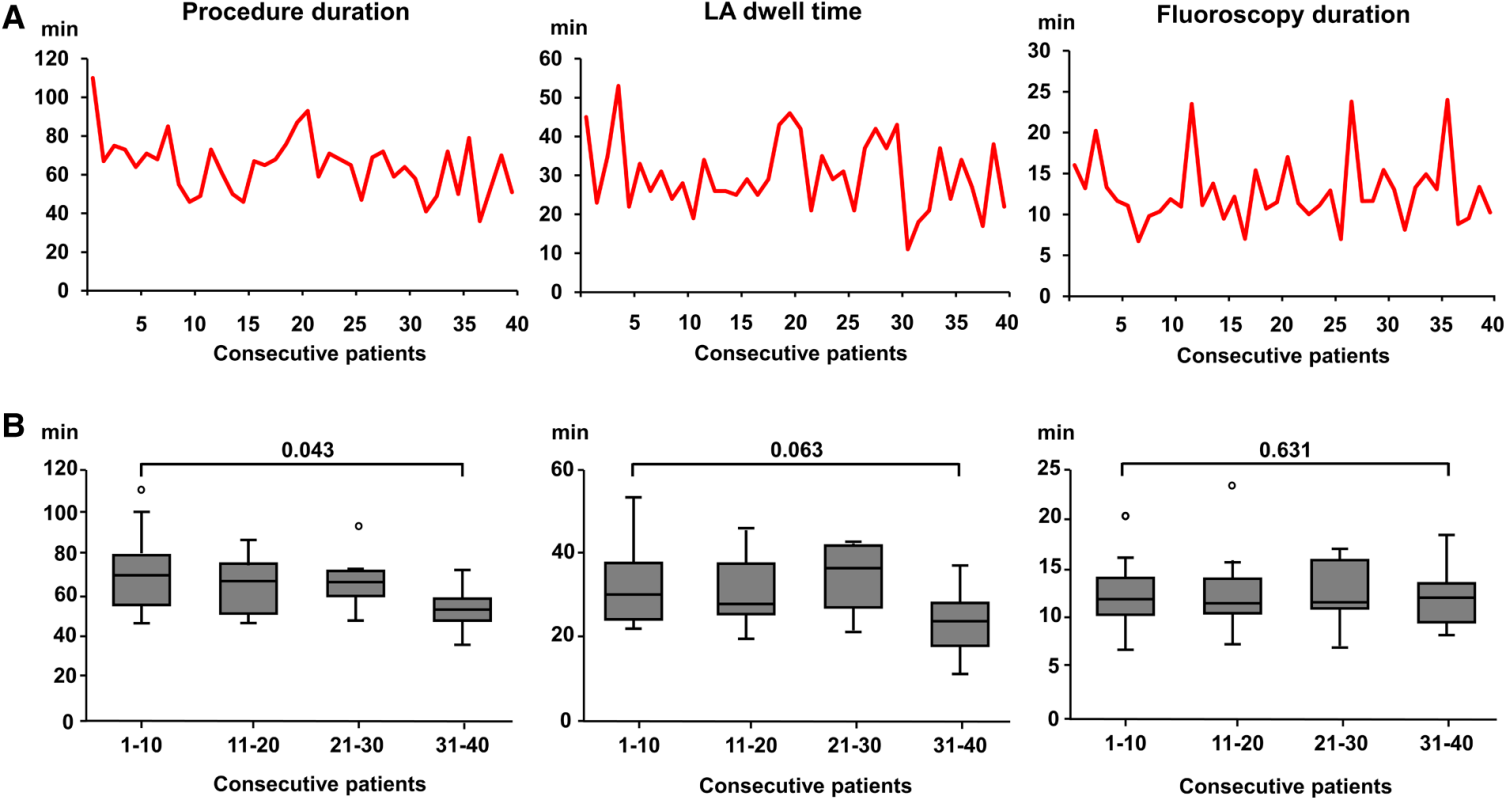
Operator learning curve with the multielectrode RF-balloon catheter. (**A**) Procedure duration (*left*), left atrial dwell time (*middle*) and fluoroscopy duration (*righ*t) with increasing operator experience in 40 consecutive patients in the HELIOSTAR™-cohort. (**B)** Boxplot of procedural parameters (see above) in the HELIOSTAR™- cohort with increasing operator experience. The cohort of consecutive patients were divided into subgroups by quartiles. The first and last 10 patients were compared, respectively. LA, left atrial.

The operator learning curve over the course of increasing experience with the device showed a certain degree of inter-procedural variability in procedural parameters, possibly influenced by technical (e.g., troubleshooting) as well as patient-specific factors (Figure 2A). Comparing the operator learning curve with respect to the first and the last 10 patients of this cohort undergoing PVI by HELIOSTAR™, there was a statistically significant decrease in overall procedure duration whereas there was no significant additional reduction in LA dwell times or fluoroscopy duration (Figure 2B).

### Complications

One female patient (age 70 years) showed acute on chronic renal failure, lactate acidosis and elevated liver enzymes ∼8 h after the procedure. In echocardiography, minimal pericardial effusion without hemodynamic significance was detected. As only 40 ml of contrast had been applied in this case during the procedure and other causes for this clinical deterioration could be excluded, it was presumed that undetected post-procedural hypotension due to possibly incorrect blood pressure measurements in this obese patient might have been the underlying cause. A pathophysiological relation to the ablation procedure could not be established. The patient was transferred to ICU for close monitoring. Both renal and metabolic function improved spontaneously after additional fluid replacement therapy. As to minor complications, not necessitating medical intervention, one patient complained of local inflammation and pain at the puncture site with spontaneous recovery after 1 week.

In-hospital arrhythmia recurrence occurred in 4 patients (10.0%) and terminated spontaneously or was cardioverted before discharge. Of note, no atrio-esophageal fistula or phrenic palsy was observed in this cohort.

## Discussion

The relevant health burden associated with AF and recent data implicating a potential prognostic benefit of early rhythm control underline the need for durable and safe therapies which can be implemented at ablation centers in a time-efficient manner (6).

This study shows that PVI with the multielectrode RF-balloon catheter could be introduced with high procedural efficacy and safety at a high-volume ablation center. Overall procedure and fluoroscopy duration were low with respect to implementation of a novel device (7). Even though procedure times were already satisfactorily low during the initial procedures performed with the RF-balloon catheter, they could be reduced even further with increasing operator training.

The multielectrode RF-balloon-catheter has been demonstrated to offer high procedural efficacy and safety in initial multicenter trials (3, 4). In comparison to these trials, the cohort analyzed in this study consisted of non-selected, real-world patients presenting for AF-ablation via everyday clinical routine. Accordingly, one third of patients suffered from persistent AF whereas previous studies evaluating this new technology only included patients with paroxysmal AF. In addition, patients in this cohort were older and more often diagnosed with cardiac co-morbidities and more progressed LA-dilation in comparison to the initial multicenter trials. This might have potentially predisposed for a higher risk of procedural complications and reduced success rates. Nevertheless, rates of successful acute PVI, single-shot isolation and short-term PV-reconnection were comparable to the previous multicenter trials in this real-world cohort. Furthermore, rates of serious procedural complications were very low and consisted of one case of acute on chronic renal failure without clear causal relation to the procedure.

In comparison to the procedural characteristics from the Multi-electrode Radiofrequency Balloon Catheter use for the Isolation of the Pulmonary Veins trial (SHINE), procedure times and LA dwell times were shorter whereas fluoroscopy duration was similar in our cohort. This may be due to further optimizations in the workflow introduced by the manufacturer since the first trials. In comparison to a recently published real-world experience from two European centers, procedure and LA dwell times were also shorter in our cohort, whereas fluoroscopy duration was higher (8). Two other recently published studies including real-world patient data from experienced, high-volume centers describe similar procedural characteristics in comparison to our data (9, 10). Some variation in procedural data between studies may be due to different center-specific approaches as to catheter visualization during mapping and ablation. Previous experience of the operator with CB-ablation may have contributed to the comparably short initial procedure times as several steps in the workflow are transferable to this technology.

In more than one in five procedures technical troubleshooting led to time delays. Necessary troubleshooting is to be expected in every new technology. In our cohort, there were no complications or relevant procedural risks associated with intermittent malfunctions of technical equipment. However, in order to reflect the real-world experience of implementation of this novel -technology at a high-volume ablation center we included time delays due to troubleshooting into the analysis of overall procedure duration and LA dwell times. Nevertheless, our study data show satisfactory procedural parameters revealing that this technology can be easily implemented at experienced ablation centers with previous experience in “single-shot”- or “over-the-wire”-techniques.

As to the operator learning curve, there was an additional decrease in procedure duration over the course of increasing operator experience, whereas LA dwell times and fluoroscopy duration showed no significant decrease. Previous CB-ablation experience and transfer of selected aspects of the workflow to ablation with the novel RF-balloon catheter may have contributed to a lack of further improvement of procedural parameters. BMI showed a weak correlation with procedure duration. The absolute difference in BMI between patients in the first and last quartiles of the cohort was small and its clinical significance can be doubted. Nevertheless, an influence of this baseline parameter in addition to the learning curve cannot be excluded.

Previous data on the implementation of a novel cryoballoon (POLARx™, Boston Scientific, St. Paul, USA) at high-volume centers also show acceptable procedure and fluoroscopy times at implementation, with a tendency of further reduction over the course of increasing operator experience (11). Additionally, in that study a reduction in complication rates with a novel CB was observed after about 25 cases. Due to the exceptionally low complication rate in our cohort we could not analyze this aspect of the learning curve. However, the low incidence of device- and ablation-related complications corresponds to previous observations from the multicenter SHINE trial. as well as to data from a large real-world study from a high-volume ablation center (3, 9). This highlights the fact that new implementation of the RF-balloon technology can be performed time-efficiently and safely at experienced AF-ablation centers.

Even though balloon-based ablation techniques have been shown to enable a steep learning curve as to procedural outcome and reduction of complications in inexperienced operators, the overall and case volume of the center plays a vital role as to efficacy and safety of AF-ablation (12, 13). In our study, the operator possessed extensive experience in both CB- and RF-based AF-ablation. However, even at high-volume centers and in experienced operators, an ongoing, long-term learning curve has been shown with respect to additional reduction in procedure duration and fluoroscopy times (14).

In a medium-term clinical follow-up of six months after the index procedure, arrhythmia recurrence rate was higher in this cohort than in previous studies on this device. This may be due to the unselected real-world patient cohort at a university hospital analyzed in this study which was characterized by higher rates of persistent AF, adverse LA remodeling and more co-morbidities in comparison to previous published cohorts of initial trails evaluating the device. Subgroup analyses of patients with and without arrhythmia recurrence revealed numerical differences in sex and rates of persistent AF, however, none of the analyzed baseline parameters showed a statistically significant difference between subgroups. Statistical analyses of predictors for arrhythmia recurrence may be limited be due to the small subgroup size.

Another real-world study of 104 patients recently reported freedom from recurrent arrhythmia of 82.9%. However, patients with persistent AF constituted the minority in this cohort and more than a third of all patients still received antiarrhythmic drug therapy at follow-up (9). A prospective, real-world observation from the AURORA collaboration showed acute procedural efficacy in a cohort with a higher rate of persistent AF (43% of patients) (10). Long-term results of this study are awaited with interest and will contribute essential evidence regarding experience with the RF-balloon from everyday clinical practice.

In the subgroup analysis of our cohort, patients with persistent AF, constituting a third of the overall cohort, showed increased LA diameters and a lower median LVEF. With respect to other baseline and procedural parameters there were no statistically significant differences, reflecting an otherwise homogenous cohort. Progressive atrial remodeling in a relevant number of patients in this study may have contributed to the higher arrhythmia recurrence rate. As 10 of 40 patients could not be contacted successfully by telephone follow-up, there may be a bias towards patients with documented arrhythmia recurrence who presented at the center due to arrhythmia symptoms. Additionally, anatomical and technical limitations in the index procedure, e.g., close proximity to the phrenic nerve or a significant esophageal temperature rise, may have contributed to medium-term PV reconnection. This was seen in single cases undergoing repeat ablation in our cohort. Potential limitations of the device as to isolation of left common ostia has to be evaluated in larger patient populations. However, long-term clinical outcome may additionally improve with increasing operator experience and should be the subject of future larger-scale studies.

With respect to the long-term outcome, therapy stratification and adequate patient selection may be crucial for technologies like CB-ablation, primarily targeting PV-dependent AF. The 3D-mapping-based substrate characterization obtained during HELIOSTAR™-procedures may provide additional patient-specific information on the arrhythmic substrate in order to individualize treatment strategies. A previous case report and a prospective cohort study describe the feasibility of ablating extra-PV targets using the multielectrode RF-balloon catheter. In the latter study, no serious complications occurred using the RF-balloon catheter for posterior wall isolation. Dedicated large studies evaluating efficacy and safety of the device for this approach would be of interest (15, 16). In case of AF-recurrence, previous information on the degree of pre-existent adverse LA-remodeling may be useful for patient counseling on individualized prognostic evaluation with respect to subsequent rhythm control strategies.

### Study limitations

The primary goal of this study was to assess the early phase of implementation of this technology in a real-world cohort. Therefore, the number of patients considered is limited. Nevertheless, a relevant learning curve as to procedure duration could be distinguished. Long-term outcome data are subject to ongoing analyses and the effect of operator experience on long-term rhythm-associated endpoints was not included in this first part of the study, focusing on immediate procedural, in-hospital and medium-term clinical outcome. The low number of patients with available follow-up data at this stage of the study constitutes an additional limitation, particularly affecting efficacy analysis. Additionally, limited cohort size may have had impact on the subgroup analysis of patients with paroxysmal or persistent AF, as clinically relevant numerical differences in arrhythmia recurrence rates failed to show statistical significance.

In the time period between initiation of the study and full market release of this technology, several additional modifications and optimizations have been introduced by the manufacturer. This includes improved visualization of the navigational LASSOSTAR™ NAV in the 3D-mapping system, which may further support reduction in fluoroscopy use and facilitate quick and safe implementation of this technology. Detection rate of ectopic PV activity was low in our cohort (42%). Quality of PV electrogram recording may additionally improve with technical optimizations of the catheters. In the majority of cases, acute PV-reconnection was excluded by assessing local electrograms at PV ostia using the LASSOSTAR™ catheter rather that repeat 3D-mapping at the end of the procedure. This may be associated with diagnostic limitations. However, it corresponds to the center’s standard protocol for other single-shot PVI-devices (e.g., cryoballoon-ablation, pulsed field ablation). Additionally, neither a prolonged intraprocedural waiting time after PVI nor adenosine application were part of the center-specific standards during for PVI, thus, dormant PV conduction may have been missed. As to the detection of oesophageal lesions or atrio-oesophageal fistula, endoscopy was not routinely performed and only scheduled for patients with clinical suspicion of these conditions.

The single-center design of the study is associated with inherent limitations as the presented data reflect the experience of only one high-volume ablation center and one operator with respect to the learning curve. However, procedural workflow was standardized and is described in detail in the Methods section for comparability of procedural data. Previously acquired expertise of the operator in LA- and CB-ablation ensured skilled performance of transseptal puncture and catheter navigation in the LA. Transferability of these results to less experienced physicians or centers is therefore limited. However, these prerequisites enhanced comparability of procedural outcomes to other technologies excluding non-device-related but rather operator-associated confounders.

## Conclusion

At a high-volume ablation center, PVI with the novel multielectrode RF-balloon catheter can be achieved with high acute procedural efficacy and safety. Previous experience and established workflows with “single-shot” ablation techniques may be beneficial for time-efficient introduction of this novel technology in clinical practice. Future, multicenter trials are needed to analyze long-term clinical outcome in larger real-world patient cohorts undergoing AF-ablation with this new technology.

## Data Availability

The datasets presented in this article are not readily available because data protection regulations apply. The data related to and generated during this study are available from the corresponding author upon reasonable request. Requests to access the datasets should be directed to maura.zylla@med.uni-heidelberg.de.
