# Supplementary material for: Implementation of the multielectrode radiofrequency-balloon in real-world clinical practice—operator learning curve and procedural outcome at a high-volume center
Source: Front Cardiovasc Med. 2023 Nov 14;10:1208250. doi: 10.3389/fcvm.2023.1208250 (PMC10682364; doi:10.3389/fcvm.2023.1208250)
Supplement: Supplementary file 1 [file Table1.docx]

**Response to Reviewers – Revision 2**

**Manuscript ID: 1208250**

**Zylla et al.: Implementation of the multielectrode radiofrequency-balloon in real-world clinical practice - operator learning curve and procedural outcome at a high-volume center**

We would like to thank the Editors and the Reviewers again for their encouraging comments and valuable suggestions, leading us to further improve our manuscript. Details are outlined below. Changes related to the second revision are marked in yellow in the revised manuscript.

**Reviewer 2:**

We thank the reviewer for the endorsement of our manuscript in the context of its re-classification under the “clinical trial”-category. The suggestions for improvement listed in the Review Forum were addressed in the first revision of the manuscript and are included in the current version.

**Reviewer 4:**

**The manuscript by Maura M. Zylla et al. aimed to evaluate the operator learning curve and procedural outcome during the implementation of the multielectrode RF-balloon at a high-volume center. It was a prospective study. 40 patients were included. 157/157 pulmonary veins (PVs) were successfully isolated, including three cases with left common PV-ostia. Median procedure duration was 62.5 min (Q1=50.0 min; Q3=70.5 min). Sixteen patients (47.1%) experienced arrhythmia recurrence, and 5 patients (14.7%) underwent repeat ablation during 6 months FU. Comparing the operator learning curve with respect to the first and the last 10 patients of this cohort undergoing PVI by HELIOSTAR^TM^, there was a statistically significant decrease in overall procedure duration, whereas there was no significant additional reduction in LA dwell times or fluoroscopy duration. The manuscript is well-written and understandable.**

We thank the reviewer for their encouraging evaluation of the manuscript and the positive feedback regarding presentation of the data. Additionally, we appreciate the suggestions for further analyses.

**Did the authors assess parameters associated with the recurrence of AF?**

We thank the reviewer for this valuable suggestion regarding analyses of predictors for arrhythmia recurrence. In response to this remark, we performed subgroup analyses between patients with and without arrhythmia recurrence with respect to baseline parameters and in-hospital recurrence as known potential predictors for AF recurrence. However, even though there were numerical differences in some parameters, e.g. rate of persistent AF or sex, none of these showed a statistically significant difference between subgroups. This may be due to the overall limited size of the subgroups. The results of the respective subgroup analyses were included in an additional table S1 in the online supplement, and are discussed in the Results and Discussion sections of the revised manuscript.

**Table S1	Subgroup analysis according to rhythm-associated outcome**

|  | **Arrhythmia recurrence (n=16)** | | **No arrhythmia recurrence**  **(n=18)** | | **P-Value** | |
| --- | --- | --- | --- | --- | --- | --- |
| **Baseline parameters** | | | | | | |
| Age, years, median (Q1;Q3) | 63.5 | (60.3;71.5) | 67.5 | (56.8;73.3) | | 0.986 |
| Male, n (%) | 13 | (81.3) | 11 | (61.1) | | 0.270 |
| Persistent AF, n (%) | 7 | (43.8) | 5 | (27.8) | | 0.475 |
| LA-diameter, mm, median (Q1;Q3) | 45.0 | (43.0;48.0) | 43.5 | (37.0;46.5) | | 0.299 |
| LVEF, %, median (Q1;Q3) | 55.0 | (40.5;59.0) | 58.0 | (54.0;60.0) | | 0.068 |
| Hypertension, n (%) | 12 | (75.0) | 17 | (94.4) | | 0.164 |
| Coronary artery disease, n (%) | 2 | (12.5) | 5 | (27.8) | | 0.405 |
| BMI, kg/m^2^, median (Q1;Q3) | 29.1 | (26.5,30.9) | 27.6 | (26.5,30.9) | | 0.512 |
| In-hospital arrhythmia recurrence, n (%) | 2 | (12.5) | 1 | (5.6) | | 0.573 |

BMI=body mass index; LA=left atrium; LVEF=left ventricular ejection fraction

**Results, page 9/10, ll. 237 ff.**

In order to detect potential predictors for arrhythmia recurrence, baseline parameters between patients with and without arrhythmia recurrence at six months were compared. There was no statistically significant difference regarding age, sex, LA diameter, type of AF, LVEF, cardiovascular risk factors, BMI or rate of in-hospital recurrence between the two subgroups.

**Discussion, page 13/14, ll. 346 ff.**

Subgroup analyses of patients with and without arrhythmia recurrence revealed numerical differences in sex and rates of persistent AF, however, none of the analyzed baseline parameters showed a statistically significant difference between subgroups. Statistical analyses of predictors for arrhythmia recurrence may be limited be due to the small subgroup size.

**The factors associated with the longer duration of the procedure? were there any? only the learning curve?**

With respect the reviewer’s suggestion we performed further subgroup and correlation analyses to detect possible association of baseline parameters with procedure duration. BMI showed a weak correlation with procedure duration. However, the absolute difference in BMI between patients in the first and last quartiles of the cohort of 1,3 kg/m2 was small and likely not clinically significant. Nevertheless, we discuss this aspect as a potential influencing factor.

**Results, page 8, ll. 199 ff.**

There was no statistically significant difference in procedure duration between sexes (P=0.192) or according to type of AF (P=0.85), and no significant correlation with LA-diameter (correlation coefficient=0.056, [CI= -0.260;0.362]). Procedure duration showed a weak correlation with BMI (correlation coefficient=0.432, [CI=0.129;0.649]) and patients in the last quartile of the cohort had a slightly lower median BMI (27.7 kg/m2 [Q1=24.2 kg/m2; Q3=30.3 kg/m2]) than patients in the first quartile (29.0 kg/m2 [Q1=26.6 kg/m2; Q3=33.1 kg/m2]), P=0.045).

**Discussion, page 12/13, ll. 317 ff.**

BMI showed a weak correlation with procedure duration. The absolute difference in BMI between patients in the first and last quartiles of the cohort was small and its clinical significance can be doubted. Nevertheless, an influence of this baseline parameter in addition to the learning curve cannot be excluded.

**What about antiarrhythmic drug after the procedure? in how many patients? how often amiodarone?**

We thank the reviewer for pointing out this outcome parameter and added this relevant aspect in the Results section.

**Results, page 8, ll. 206ff.**

Nine patients continued antiarrhythmic medication (26.5%), of which three patients received amiodarone (8.8%).
